# Cytomegalovirus (CMV) immune monitoring with ELISPOT and QuantiFERON-CMV assay in seropositive kidney transplant recipients

**DOI:** 10.1371/journal.pone.0189488

**Published:** 2017-12-12

**Authors:** Hyeyoung Lee, Ki Hyun Park, Ji Hyeong Ryu, Ae-Ran Choi, Ji Hyun Yu, Jihyang Lim, Kyungja Han, Sang Il Kim, Chul Woo Yang, Byung Ha Chung, Eun-Jee Oh

**Affiliations:** 1 Department of Laboratory Medicine, Seoul St. Mary’s Hospital, College of Medicine, The Catholic University of Korea, Seoul, Korea; 2 SamKwang Medical Laboratories, Seoul, Korea; 3 Department of Biomedical Science, Graduate School, College of Medicine, The Catholic University of Korea, Seoul, Korea; 4 Transplant Research Center, Seoul St. Mary’s Hospital, College of Medicine, The Catholic University of Korea, Seoul, Korea; 5 Division of Nephrology, Department of Internal Medicine, Seoul St. Mary’s Hospital, College of Medicine, The Catholic University of Korea, Seoul, Korea; 6 Division of Infection, Department of Internal Medicine, Seoul St. Mary’s Hospital, College of Medicine, The Catholic University of Korea, Seoul, Korea; Fujita Health University, School of Medicine., JAPAN

## Abstract

Although cytomegalovirus (CMV) specific cell-mediated immunity (CMI) has been suggested as a predictive marker for CMV infection, proper CMI monitoring strategy in CMV-seropositive recipients and optimal method are not defined. The aim of this study was to evaluate two interferon gamma release assays during early post-transplant period as a predictor of the development of CMV infection in CMV-seropositive patients. A total of 124 CMV-seropositive recipients who received kidney transplantation from CMV-seropositive donor were prospectively examined. At pre-transplant and post-transplant 1 and 3 months, CMV-CMIs were tested using QuantiFERON-CMV assay (QF-CMV) and CMV specific T cell ELISPOT against CMV pp65 and IE-1 antigens (pp65-ELISPOT, IE-1-ELISPOT). CMV DNAemia occurred in 16 (12.9%) patients within 3 months after transplant. Post-transplant pp65 or IE-1 ELISPOT response, but not QF-CMV, was significantly associated with CMV DNAemia. The pp65 ELISPOT (cut-off; 30 spots/200,000 cells) and IE-1 ELISPOT (10 spots/200,000 cells) at post-transplant 1 month predicted the risk of post-transplant CMV DNAemia (*P* = 0.019). Negative predictive values (NPV) for protection from CMV DNAemia in case of positive ELISPOT results were 94.5% (95% CI: 86.9–97.8%) and 97.6% (95% CI: 86.3–99.6%) in pp65-ELISPOT and IE-1-ELISPOT assays, respectively. These results suggest that the variability may exist between CMV ELISPOT assays and QF-CMV, and CMV ELISPOT at post-transplant 1 month can identify the risk of CMV DNAemia in seropositive kidney transplant recipients.

## Introduction

Despite advances in management strategies, cytomegalovirus (CMV) infection remains one of the most common and serious complications in kidney transplant recipients [1]. The risk of CMV infection or reactivation can be predicted by pretransplant CMV serostatus and immunosuppressive regimes. CMV-seropositive recipients are at moderate risk of CMV infection, and universal prophylaxis or preemptive ganciclovir is recommended based on monitoring for CMV DNAemia [2]. However, the consensus has not yet been established about the cut off CMV viral load indicating preemptive antiviral therapy. Risk factors requiring CMV prophylaxis in seropositive recipients have not been defined either.

In recent years, several reports have suggested that measurement of CMV specific T cell activity might reflect patients’ ability to control the virus and predict the risk for post-transplant viral replication [3–10]. Therefore, immunologic monitoring of CMV specific T cell immunity might be an effective strategy to address this concern [2, 11]. Interferon-γ (IFN-γ) has been revealed to play a critical role in controlling CMV infection. Therefore, measurement of IFN-γ release might be a useful immune biomarker for CMV infection. QuantiFERON-CMV assay (QF-CMV, Cellestis, a QIAGEN Company, Australia) is a commercially available enzyme-linked immunosorbent assay to detect IFN-γ released in whole blood by ex vivo stimulation with human leukocyte antigen (HLA) class I restricted CMV peptides from pp28, pp50, pp65, IE-1, IE-2, and gB [6]. IFN-γ can also be measured by enzyme-linked immunospot (ELISPOT) assay to assess T cell immune activity by measuring IFN-γ production following stimulation with CMV antigens such as phosphoprotein 65 (pp65) or immediate early 1 (IE-1).

In this study, kidney transplanted patients were serially monitored at three time points (pretransplant, post-transplant 1 month, and post-transplant 3 months) via two IFN-γ release assays: QF-CMV and CMV ELISPOT assays (against CMV pp65 and IE-1 antigens). Although both assays measure IFN-γ to detect T cell response upon stimulation by CMV antigens, their characteristics in methods (ELISA vs. ELISpot) and principles (CMV-specific CD8+ vs. CD4+ plus CD8+ responses, respectively) are different. In addition, clinical use of CMI is still limited and proper CMI monitoring strategy in CMV-seropositive recipients remains to be elucidated. Therefore, the aim of this study was to compare and evaluate if monitoring of CMV-CMI using QF-CMV or CMV ELISPOT assays could predict CMV infection in seropositive kidney transplant recipients.

## Materials and methods

### Patients

A total of 124 CMV seropositive patients (R+) who received kidney transplant from seropositive donor (D+) at Seoul St. Mary’s Hospital from February 2014 to March 2016 were enrolled in this study. Patients were serially monitored for CMV DNAemia. CMV-CMI assays were performed for three consecutive time points: pretransplant, post-transplant 1 month, and post-transplant 3 months. Routine surveillance for CMV infection was performed based on CMV DNAemia which was monitored pretransplantation, every week for 1 month after transplantation, monthly thereafter up to 3 months post-transplantation, and then every 3 months up to one year post-transplantation. Episode of CMV DNAemia was defined by the detection of CMV DNA in recipient’s whole blood (> 3,250 IU/mL). CMV DNAemia was detected in EDTA whole blood samples using AccuPower^®^ CMV Quantitative PCR Kit (Bioneer, South Korea) through real-time polymerase chain reaction which was calibrated to World Health Organization International Standard for Human CMV. The lowest detection limit corresponded to 117 IU/mL using 200 μL of whole blood. This study was approved by the Institutional Review Board of Seoul St. Mary’s Hospital (Approval No. KC13TISI0697). All patients provided written informed consent. None of the transplant donors were from a vulnerable population and all donors or next of kin provided written informed consent that was freely given.

### Immune suppressant regimen and CMV viral treatment

Typical immunosuppressive regimen at our center has been described previously [12, 13]. Briefly, tacrolimus or cyclosporine was administered in combination with mycophenolate mofetil and prednisolone. Anti-thymocyte globulin (ATG) or anti-CD25 monoclonal antibody (basiliximab) was administered as induction therapy. In patients with donor specific HLA antibodies or in ABO incompatible kidney transplantation, only tacrolimus was administered as the main regimen. CMV infection was defined as the detection of CMV DNA in samples of whole blood. It was managed according to the standard protocol of our institution which consisted of prophylaxis and preemptive strategy. The prophylaxis therapy was performed in high risk recipients with ATG induction therapy. Intravenous ganciclovir was administered during ATG injection which ranged from 5 to 7 days at a dose of 2.5mg/kg twice a day. The dose was modified according to graft function of each patient [14]. When viral load of CMV exceeded 13,000 IU/mL, IV ganciclovir therapy was used.

### QuantiFERON-CMV assay

Freshly isolated whole blood was collected in three QF-CMV tubes (Mitogen, Nil and CMV stimulus) (1ml per tube) and incubated at 36°C overnight in an incubator with 5% CO_2_. Each tube was centrifuged at 2,000g for 15 minutes. Plasma samples were then collected and stored at -70°C until analysis for secreted IFN-γ. QF-CMV ELISA (QF-CMV, Cellestis, a QIAGEN Company, Australia) was performed to measure secreted IFN-γ levels according to manufacturer’s instructions. The recommended cut off value for QF-CMV reactivity was 0.2 IU/mL according to the manufacturer’s instruction. Data of undetermined samples (those not responding to positive control) were analyzed as negative results.

### CMV ELISPOT

CMV ELISPOT was performed using Human IFN-γ ELISPOT Ready-SET-Go^®^ kit (eBioscience, San Diego, CA, USA). Briefly, 96-well ELISPOT plates (Millipore, Cat. No. MAIPS4510) were washed and coated with a mouse monoclonal anti-human IFN-γ capture antibody overnight at 4°C. Peripheral blood mononuclear cells (PBMC) were freshly isolated from heparinized whole blood using Ficoll density gradient as described previously [13, 15]. PBMCs (2 x 10^5^cells/well) were stimulated with phorbol 12-myristate 13-acetate (PMA) / ionomycin (positive control), complete media (negative control), and CMV peptide (1 μg/mL of IE-1 or pp65, JPT Peptides Technologies, Berlin, Germany) (tests) at 36°C overnight in an incubator with 5% CO_2_. Peptides pools consist of 138 peptides (peptide scan 15mers with 11 aa overlap) through 65 kDA pp65, and 120 peptides (peptide scan 15mers with 11 aa overlap) through 55 kDA IE-1. ELISPOT using these mixtures of overlapping peptides simultaneously detect both CD4+ and CD8+ T cell responses regardless of HLA types [16]. Further process was performed according to the manufacturer’s instructions. Resulting spots were counted with a computer-assisted ELISPOT image analyzer (Cellular Technology, Cleveland, OH, USA). Results were calculated as mean values of spots/2 x 10^5^ cells PBMCs based on duplicate or triplicate measurements after subtracting response of negative control wells.

### Statistical analysis

Statistical analyses were performed using MedCalc version 15.5 (MedCalc, Mariakerke, Belgium). Categorical variables were compared by Pearson uncorrected Chi-square test while continuous variables were compared using Mann-Whitney U test. Receiving operator curve (ROC) analysis plots were used to define cut-off number of spots for pp65- and IE-1-ELISPOT assay to predict CMV DNAemia. All reported *P* values were two-sided. Statistical significance was considered at *P* < 0.05. Kaplan—Meier curves were produced to reveal the probability of CMV DNAemia according to CMV ELISPOT positivity.

## Results

### Patient characteristics

Demographic characteristics of recipients included in this study are summarized in Table 1. Of 124 D+/R+ CMV transplants, sixteen (12.9%) recipients developed CMV DNAemia within 3 months post transplantation. Of these sixteen patients, fifteen (93.8%) experienced spontaneous clearance of DNAemia with discontinuation of mycophenolate mofetil. Only one patient who had CMV DNAemia > 320,000 IU/mL received antiviral treatment. There were no significant differences in baseline characteristics between recipients with CMV DNAemia (+) and those without CMV DNAemia (-) except donor type (Table 1). CMV DNAemia was more prevalent in deceased donor compared to that in living donor.

**Table 1:** Baseline characteristics of patients.

Characteristics	Total	CMV DNAemia (+)	CMV DNAemia (-)	P value
	(n = 124)	(n = 16)	(n = 108)	
Age at Transplantation (year)	46.0 +/-12.0^a^	48.6 +/-10.4	45.0 +/-12.2	NS
Gender; male, n (%)	65 (52.4)	10 (62.5)	55 (50.9)	NS
Re-transplantation, n (%)	22 (17.8)	3 (18.8)	19 (17.6)	NS
Deceased donor, n (%)	27 (21.8)	7 (43.8)	20 (18.5)	0.023
ABO incompatible, n (%)	20 (16.1)	1 (6.3)	19 (17.6)	NS
Desensitization, n (%)	44 (35.5)	4 (25.0)	40 (37.0)	NS
Immune suppressant, n (%)				
Cyclosporin	62 (50.0)	10 (62.5)	52 (48.1)	NS
Tacrolimus	62 (50.0)	6 (37.5)	56 (51.9)	NS
Induction therapy, n (%)				
ATG	18 (14.5)	3 (18.8)	15 (13.9)	NS
Basiliximab	106 (85.5)	13 (81.3)	93 (86.1)	NS
Serum Cr at transplantation	8.0+/-2.7^a^	8.6 +/- 2.9	7.9+/-2.7	NS
Rejection, n (%)	20 (16.1)	3 (18.8)	17 (15.7)	NS
DSA at pre-KT, n (%)	12 (9.7)	1 (6.3)	11 (10.2)	NS
HLA mismatch number, mean	3.2+/-1.7^a^	3.8 +/- 1.4	3.2+/-1.7	NS
Primary renal disease, n (%)				
Chronic glomerulonephritis	41 (33.1)	4 (25.0)	37 (34.3)	NS
DM	23 (18.5)	4 (25.0)	19 (17.6)	NS
HTN	11 (8.9)	1 (6.3)	10 (9.3)	NS
ADPKD	5 (4.0)	0 (0.0)	5 (4.6)	NS
Unknown	44 (35.5)	7 (43.8)	37 (34.3)	NS

^a^mean +/- standard deviation; NS: not significant (P > 0.05).

Abbreviations: HLA, human leukocyte antigen; DSA, Donor specific HLA antibodies; ATG, Anti-thymocyte globulin; DM, Diabetes mellitus; HTN, Hypertension; ADPKD, Autosomal dominant polycystic kidney disease; NS, non significant

P value: Categorical variables were compared by Pearson uncorrected Chi-square test and continuous variables were compared using Mann-Whitney U test.

### QuantiFERON-CMV assay

**Fig 1:**
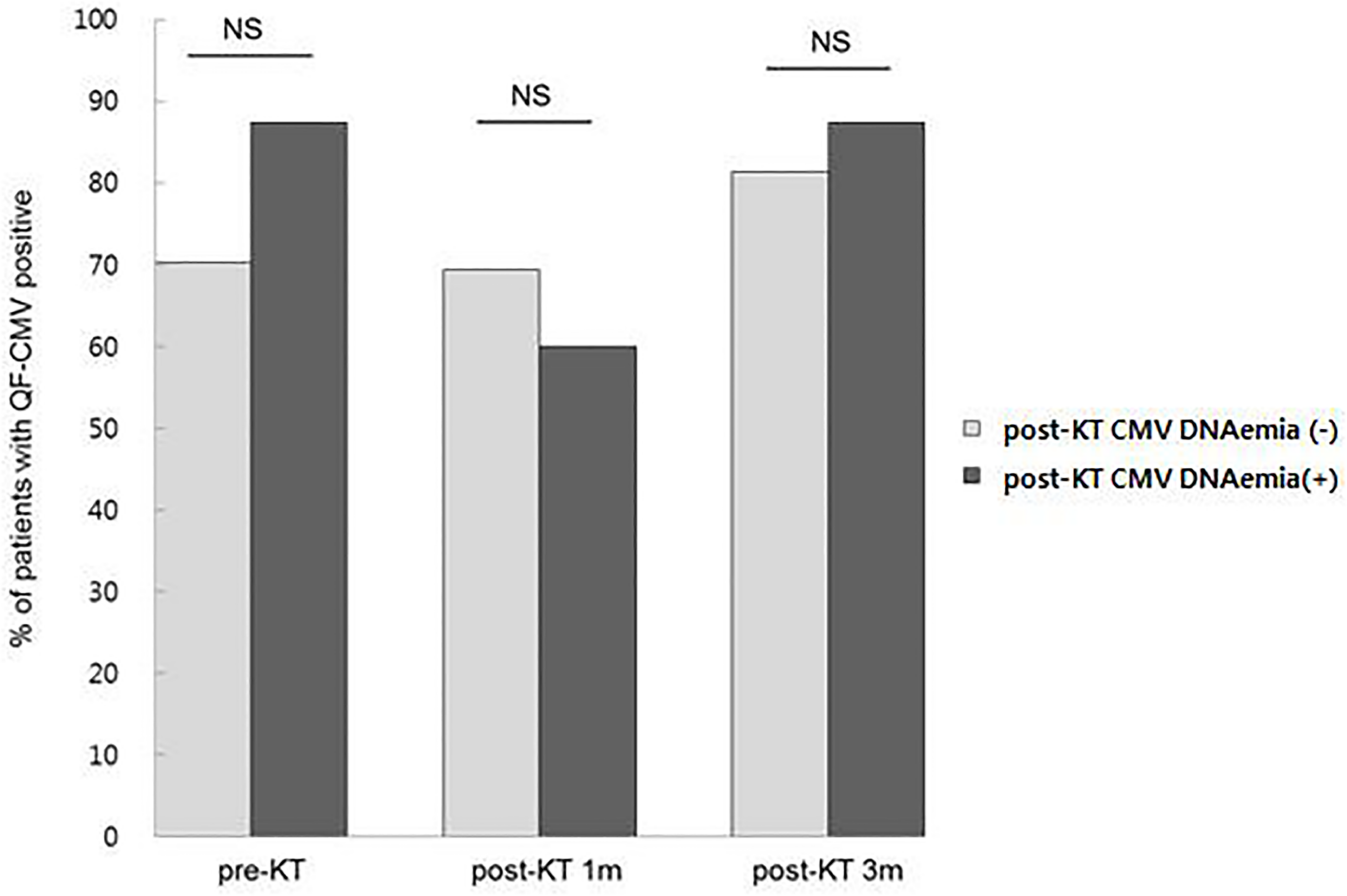
QF-CMV results at pretransplant, post-transplant 1 month, and post-transplant 3 months in recipients with post-KT CMV DNAemia (-) and CMV DNAemia (+) by Pearson uncorrected Chi-square test. There was no significant difference in frequencies of QF-CMV positivity between two groups. NS, not significant (*P* > 0.05).

Of 124 seropositive recipients, 34 (27.4%) were QF-CMV nonreactive (IFN-γ < 0.2 IU/mL) at pretransplant. There was no significant (*P* > 0.05) difference in QF-CMV positivity over the study period. Frequencies of QF-CMV positivity at three consecutive time points over the study period are shown in Fig 1. A total of 108 patients had no CMV DNAemia while 16 patients developed CMV DNAemia within 1 year post-transplant (Fig 1). Among these 16 recipients who had CMV DNAemia, 2 (12.5%), 6 (37.5%), and 2 (12.5%) showed QF-CMV (-) at pretransplant, post-transplant 1 month, and post-transplant 3 months, respectively. For the two patients who had QF-CMV (-) at pretransplant, QF-CMV (+) was found at post-transplant 1 month with CMV DNAemia development. QF-CMV results at the three consecutive time points were not statistically associated with CMV DNAemia (*P* > 0.05). QF-CMV at post-transplant 1month revealed a maximum area under the curve (AUC) of 0.552 (95% CI: 0.444–0.565, *P* = 0.601).

### CMV ELISPOT

**Fig 2:**
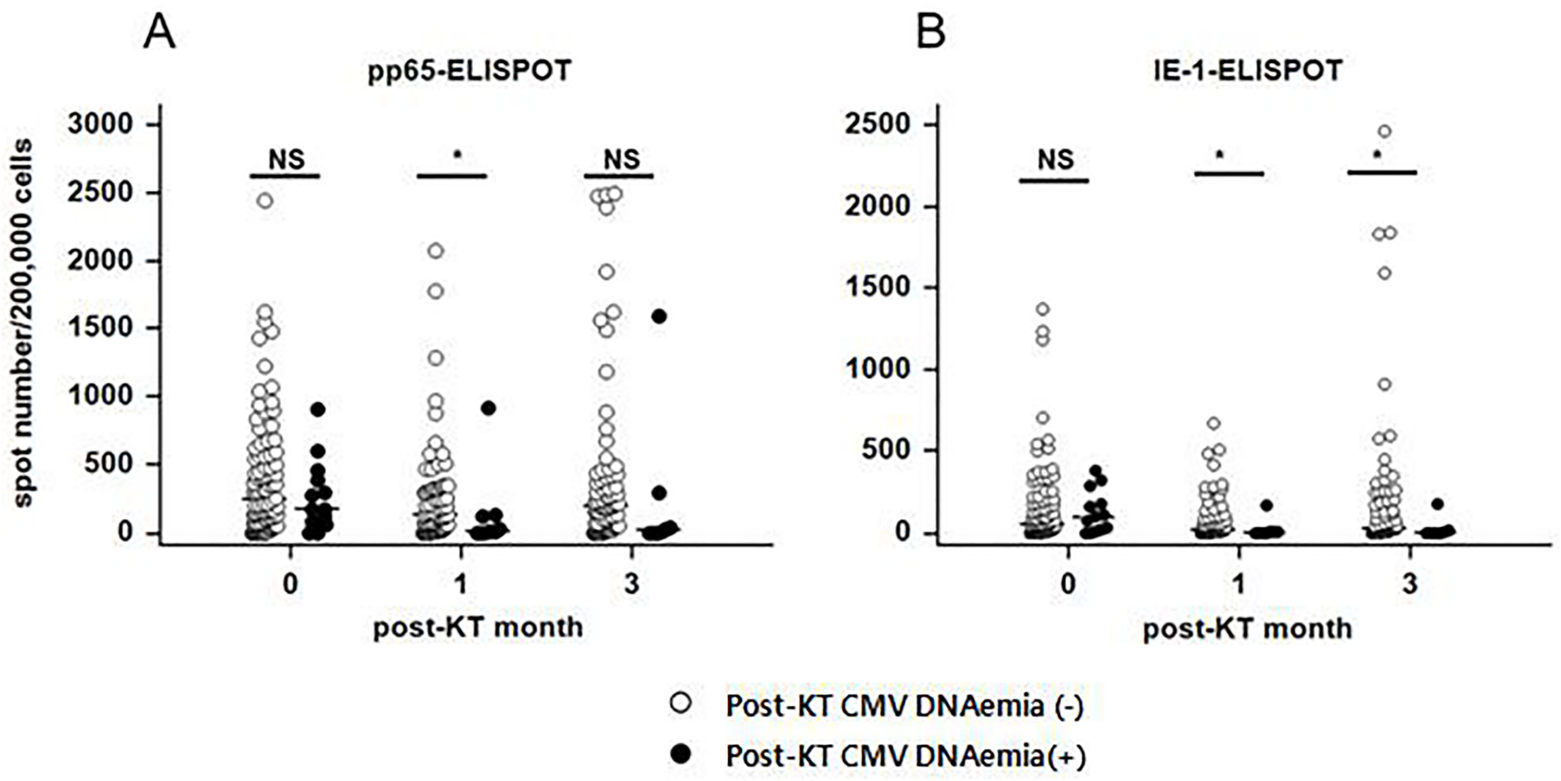
Results of pp65- and IE-1 specific ELISPOT at pretransplant, post-transplant 1 month, and post-transplant 3 months in recipients with post-KT CMV DNAemia (-) and CMV DNAemia (+) by Mann-Whitney U test. (A) For pp65-ELISPOT, patients with CMV DNAemia (+) had significantly lower response at post-transplant 1 month than patients with CMV DNAemia (-) [median (95% CI): 8.5 (1.4–130.5) vs. 138.0 (87.2–214.7), *P* = 0.016]. (B) For IE-1-ELISPOT, CMV DNAemia (+) patients showed significantly lower response at post-transplant 1 and 3 months compared to CMV DNAemia (-) patients (4.0 (1.0–8.1) vs. 18.0 (8.0–44.0), *P* = 0.019 and 2.0 (0.0–46.6) vs. 27.0 (13.0–56.4), *P* = 0.014, respectively).

Serial ELISPOT results based on pp65 and IE-1 antigens are shown in Fig 2. For pp65-ELISPOT, patients with CMV DNAemia (+) (n = 16) had significantly lower responses at post-transplant 1 month than those with CMV DNAemia (-) (n = 108) [median (95% CI): 8.5 (1.4–130.5) and 138.0 (87.2–214.7), respectively, *P* = 0.016]. For IE-1-specific ELISPOT, CMV DNAemia (+) patients showed significantly lower responses at post-transplant 1 month and 3 months compared to those with CMV DNAemia (-) (*P* = 0.019 and *P* = 0.014, respectively). Analysis of receiver operating characteristic (ROC) curve for pp65-ELISPOT and IE-1-ELISPOT assays for predicting CMV DNAemia (+) revealed maximum areas under the curve (AUC) of 0.737 (95% CI: 0.628–0.828, *P* = 0.011) and 0.729 (95% CI: 0.622–0.820, *P* = 0.004), respectively.

**Fig 3:**
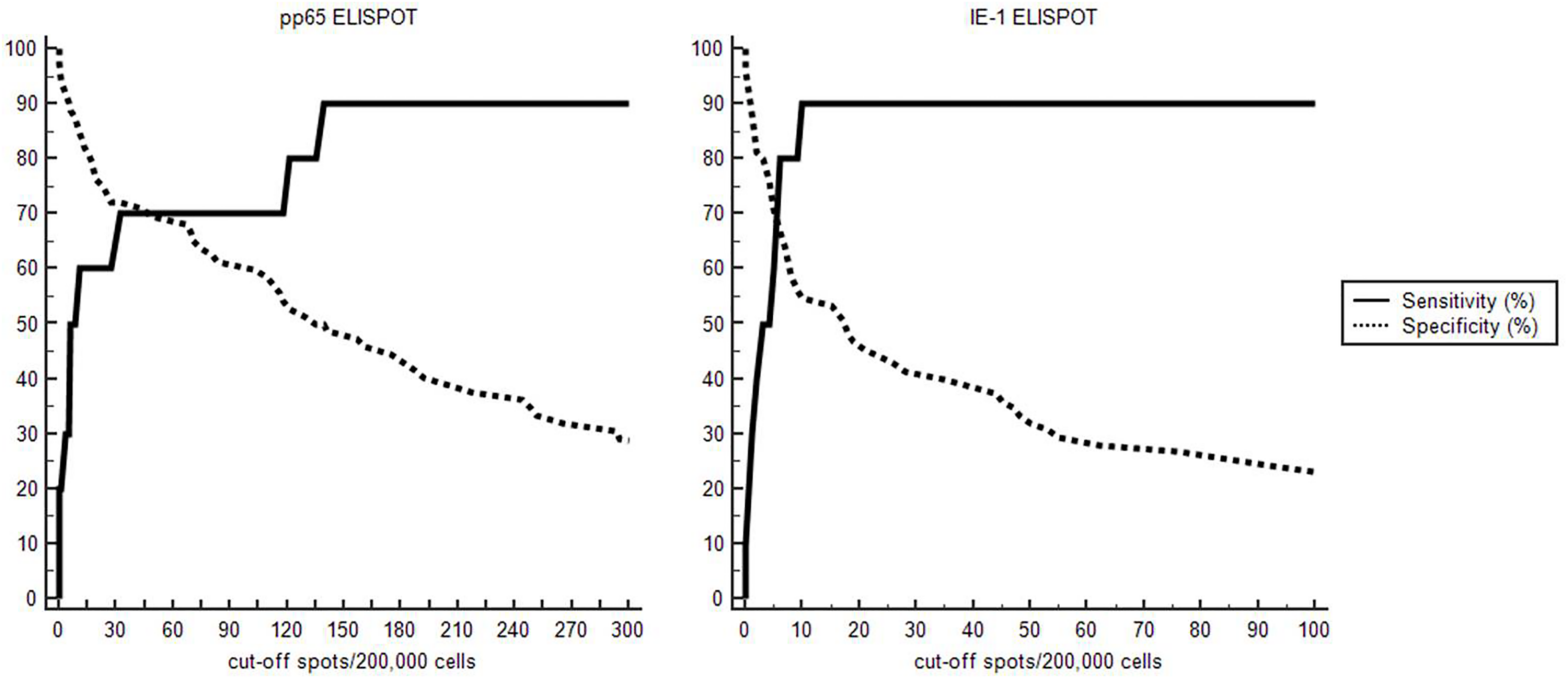
Plot versus criterion value curves for the post-transplant 1 month pp65-ELISPOT and IE-1-ELISPOT assay for predicting CMV DNAemia. The X-axis shows the cut-off spots/200,000 cells and the Y-axis shows the percentage of CMV DNAemia development. The sensitivity and specificity of pp65-ELISPOT at post-transplant 1 month for predicting CMV DNAemia were 70.0% (95% CI: 34.8–93.3) and 72.2% (95% CI: 60.4–82.1%) with a cut-off value of ≤ 30 spots/200,000 cells. IE-1-ELISPOT showed a sensitivity of 90.0% (95% CI: 55.5–99.7%) and a specificity of 54.7% (95% CI: 42.7–66.2%) to predict CMV DNAemia with a cut-off value of ≤ 10 spots/200,000 cells.

Using plot versus criterion values analysis of ROC curve, optimal cut-off values were defined as 30 spots/200,000 cells for pp65-ELISPOT and 10 spots/200,000 cells for IE-1-ELISPOT (Fig 3). With optimal cut-off values at post-transplant 1 month, their diagnostic accuracies for predicting posttransplant CMV DNAemia were assessed. The sensitivity and specificity of pp65-ELISPOT with a cut-off value of < 30 spots/200,000 cells were 70.0% (95% CI: 34.8–93.3) and 72.2% (95% CI: 60.4–82.1%) for predicting CMV DNAemia, respectively. IE-1-ELISPOT showed a sensitivity of 90.0% (95% CI: 55.5–99.7%) and a specificity of 54.7% (95% CI: 42.7–66.2%) to predict CMV DNAemia with a cut-off value of ≤ 10 spots/200,000 cells. Negative predictive values (NPV) of pp65-ELISPOT and IE-1-ELISPOT assays were 94.5% (95% CI: 86.9–97.8%) and 97.6% (95% CI: 86.3–99.6%), respectively. However, positive predictive values (PPV) were 25.9% (95% CI: 16.8–37.8%) and 20.9% (95% CI: 16.1–26.8%) in pp65-ELISPOT and IE-1-ELISPOT assays, respectively (Table 2).

**Table 2:** Diagnostic accuracy of post-transplant 1 month CMV ELISPOT assay for predicting CMV DNAemia with ROC analysis.

	Sensitivity (95% CI)	Specificity (95% CI)	PPV (95% CI)	NPV (95% CI)
pp65	70.0% (34.8–93.3)	72.2% (60.4–82.1)	25.9% (16.8–37.8)	94.5% (86.9–97.8)
IE-1	90.0% (55.5–99.7)	54.7% (42.7–66.2)	20.9% (16.1–26.8)	97.6% (86.3–99.6)

Abbreviations; PPV, positive predictive value; NPV, negative predictive value, CI, confidence interval

**Fig 4:**
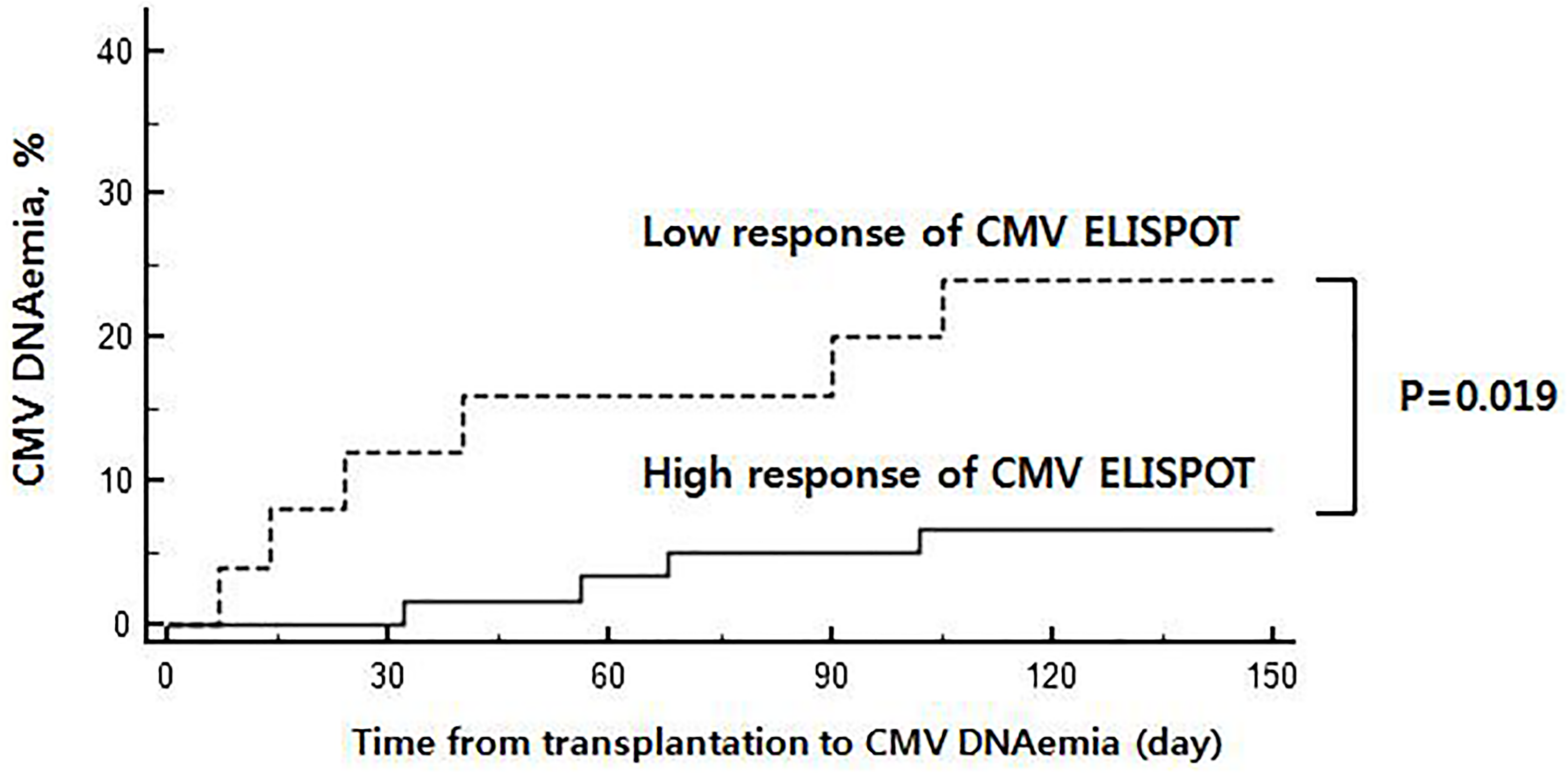
Kaplan–Meier analysis according to the CMV ELISPOT response. Kaplan-Meier failure estimate showing that patients with high response of CMV ELISPOT at post-transplant 1 month had lower development of CMV DNAemia than patients with low response of CMV ELISPOT (*P* = 0.019). Patients were classified into high response (if the either pp65 or IE-1 antigens had spot counts more than the cut-off thresholds) and low response CMV (if level of both antigens were below the cut-off thresholds) post-KT 1 month CMV ELISPOT assay.

We classified patients into high response group (if either pp65 or IE-1 ELISPOT showed positive result using optimal cut-off levels) and low response group (if both pp65 and IE-1 ELISPOT showed the negative results (< cut-off) at post-KT 1 month). Kaplan-Meier analysis revealed that patients with high response at post-transplant 1 month had lower development of CMV DNAemia than patients with low response (*P* = 0.019) (Fig 4). In subgroup analysis for patients who underwent basiliximab induction therapy (n = 106), the patients with CMV DNAemia (+) had lower ELISPOT counts at post-KT 1month compared to those in CMV DNAemia (-) [pp65-ELISPOT: 8.5 (0.0–291.0) vs. 157 (104.8–23.7.2) spots/200,000 cells, *P* = 0.0435; IE-1-ELISPOT: 3.5 (0.8–40.7) vs. 18.0 (8.0–44.9) spots/200,000 cells, *P* = 0.0325)].

## Discussion

The present study evaluated the potential of using QF-CMV and CMV ELISPOT assays to identify patients at risk of CMV infection in seropositive kidney transplant recipients. A total of 124 CMV-seropositive recipients were analyzed. Our results revealed that CMV ELISPOT assay could identify patients at risk of CMV DNAemia in seropositive kidney transplant recipients. QF-CMV assay results at three time point (pretransplant, post-KT 1 and 3 months) were not significantly different between patients with CMV DNAemia (+) and patients with CMV DNAemia (-). However, positive CMV ELISPOT at post-transplant 1 month predicted lower risk of early CMV DNAemia after transplantation. These findings suggest that CMV DNA monitoring with CMV ELISPOT at post-transplant 1 month could be helpful for making decisions on antiviral treatment during preemptive strategy.

Previous studies have demonstrated that pretransplant CMV ELISPOT responses in seropositive recipients might be useful for predicting the risk for post-transplant CMV infection [5, 17–19]. CMV ELISPOTs have been proved to be more effective in differentiation of primarily CMV infected pregnant women compared to QF-CMV [20]. Induction and maintenance immunosuppressive therapy could decrease the levels of immune competent T cells during early after transplantation. In addition, several factors can influence immunity to CMV, including acute rejection, delayed graft function, susceptibility to immunosuppressive drugs, exposure to virus antigen, and other factors not yet elucidated [21]. Our findings support results of previous reports showing that posttransplant monitoring of CMV specific T cells may play an important role in controlling CMV reactivation because it is more effective in predicting CMV infection than pretransplant measurement of CMI [4, 8]. Using ROC analysis, we demonstrated that cutoff levels of 30 spots/200,000 cells in pp65-ELISPOT and 10 spots/200,000 cells in IE-1-ELISPOT at post-transplant 1 month were able to predict CMV DNAemia with good sensitivities (70.0% and 90.0%, respectively) and specificities (72.2% and 54.7%, respectively). The presence of positive CMV ELISPOTS response at post-transplant 1 month was protective against the development of early CMV DNAemia (94.5% and 97.6% in pp65- and IE-1- ELISPOT assays, respectively). This finding was similar to previous data demonstrating that monitoring CMV specific ELISPOT and eGFR in the first month post kidney transplant could identify patients at high risk of CMV infection [4]. Although CMV prophylaxis is the most effective one to reduce CMV disease, optimal use of this approach is controversial due to drug toxicity and high cost of anti-viral agents [22]. Preemptive therapy has been thought to be effective in patients at moderate risk of CMV disease with potential advantages, including reduced antiviral drug toxicity and cost-effectiveness [4]. For optimal preemptive therapy, there is a consensus that viral load should be monitored every week for 3 to 4 months after transplantation with meticulous weekly monitoring for preemptive therapy [2]. However, its high cost is a limiting factor. In addition, major risk associated with preemptive therapy strategy is nonadherence to regular PCR monitoring. It might lead to severe CMV disease. In addition, there is no universally accepted CMV viral load for starting preemptive therapy [23]. Therefore, CMV ELISPOT results could be helpful for selecting effective preemptive antiviral strategy, supporting results of previous reports that both immune response and DNAemia should be monitored while on preemptive therapy to determine the risk of infection [2, 11, 24].

Cell-mediated immunity is considered the primary defense mechanism to control CMV reactivation. Both CD4+ and CD8+ T cells are involved in the defense against CMV infection with production of interferon-γ [25]. For QF-CMV and CMV ELISPOT assays, although both are IFN-γ release assays, several features of their methods and principles are different. QF-CMV measures IFN-γ production in defined volume of whole blood (1 ml) by ex vivo stimulation with HLA restricted CMV peptides whereas ELISPOT assay is made on a given number of PBMCs isolated from peripheral blood [3]. QF-CMV uses 22 peptides from pp28, pp50, pp65, IE-1, IE-2, and gB, whereas the ELISPOT uses overlapping peptide pools from only IE-1 or pp65. Moreover, QF-CMV detects only CD8 T cell responses whereas CMV ELISPOT assay can detect both CD4 and CD8 T cell responses against CMV specific proteins. Although multiple CMV specific proteins for T cell response exist, pp65 and IE-1 have each been identified as the predominant target of CMV-specific T cell responses [26–30]. Previous reports have shown high frequencies of pp65 or IE-1 specific cytotoxic T cell precursors in CMV seropositive individuals [31–34]. ELISPOT assay directly recognize virus-specific cytotoxic T lymphocytes, thus it may reflect T cell specificities in vivo [35]. The peptides used in ELISPOT were complete protein-spanning mixtures of overlapping entire protein sequence and detected T-cell responses regardless of HLA type [16]. In contrast, QF-CMV consisted in several HLA-restricted peptides, and patients with uncommon HLA types might be not detected correctly in this assay. The use of different peptide pool combination might influence T cell responses. In addition, HLA type might affect the efficiency of antigen presentation and CMV-CMI measured [36]. Finally, QF-CMV technology is based on ELISA, which measures the level of secreted IFN-γ and thus presents a lower sensitivity of detection than the ELISpot assay, which measures the number of IFN-γ-secreting cells.

In terms of QF-CMV, 27.4% of enrolled recipients were nonreactive at pretransplant. Positive-QF-CMV results were associated with high pp65- or IE-1- ELISPOT values. However, we could not find meaningful results of QF-CMV for predicting CMV DNAemia. This result is in contrast to a previous report showing that both ELISPOT and QF-CMV assays have similar abilities to predict CMV infection [3]. As QF-CMV measured T cell responses by ex vivo stimulation with certain HLA restricted CMV peptides, patients with uncommon HLA types might not be detectable in current QF-CMV assay [3]. This could be a reason for false negative QF-CMV results. Further studies are needed to confirm our findings using large number of CMV-seropositive recipients.

This study has some limitations. Most CMV viremic cases showed benign clinical course. They were successfully cleared without anti-viral agents. Therefore, our results could not show whether monitoring of CMV-CMI can differentiate cases that will show unfavorable outcomes in terms of CMV DNAemia. This may limit the clinical usefulness of CMV-CMI monitoring. Despite these limitations, our study focused on CMV-seropositive recipients and presented sequential CMI results with two different IFN-γ releasing assays and demonstrated diagnostic value of CMV ELISPOT assay at post-transplant 1 month. Further studies are needed to confirm our results and validate those cutoff levels of CMV ELISPOT assays.

In conclusion, pp65- and IE-1 specific ELISPOT assays at post-transplant 1 month can be used to identify patients at risk of CMV DNAemia in seropositive kidney transplant recipients.
